# Vitamin D Nutritional Status and Its Related Factors for Chinese Children and Adolescents in 2010–2012

**DOI:** 10.3390/nu9091024

**Published:** 2017-09-15

**Authors:** Yichun Hu, Jing Chen, Rui Wang, Min Li, Chunfeng Yun, Weidong Li, Yanhua Yang, Jianhua Piao, Xiaoguang Yang, Lichen Yang

**Affiliations:** Key Laboratory of Trace Element Nutrition of National Health and Family Planning Commission, National Institute for Nutrition and Health, Chinese Center for Disease Control and Prevention, Beijing 100050, China; huyichun0319@163.com (Y.H.); chenjing1909@sina.com (J.C.); bluingwang@126.com (R.W.); lmlovely@163.com (M.L.); sallyycf@163.com (C.Y.); weidongli0507@126.com (W.L.); yhyang@163.com (Y.Y.); piaojh@163.com (J.P.); xgyangcdc@vip.sina.com (X.Y.)

**Keywords:** vitamin D, Chinese children and adolescents, 25-hydroxyviramin D

## Abstract

Vitamin D plays a critical role in calcium and phosphate metabolism and helps maintain skeletal integrity in childhood, yet vitamin D status in Chinese children and adolescents is not well documented. The aim of this study was to assess the vitamin D status and analyze the risk factors for vitamin D deficiency in Chinese children and adolescents aged 6–17 years. Serum 25 hydroxyvitamin D (25(OH)D) was measured with a radioimmunoassay kit in 15,000 children and adolescent participants in the Chinese national nutrition and health survey (CNNHS) 2010–2012. Age, gender, region type, ethnicity, outdoor time, and vitamin D supplementation were recorded in unified design questionnaires. The season was recorded by the date of blood taken; location was divided into north and south by China’s Qinling Mountains and Huaihe River; and ambient ultraviolet B (UVB) radiation level was classified according to the corresponding dose of each participant living area from National Aeronautics and Space Administration data. 14,473 participants from the cross-sectional study of CNNHS 2010–2012 were included in this study. The median serum 25(OH)D concentration was 48.2 (35.4–63.4) nmol/L, and the concentration for males was 50.0 (36.5–65.7) nmol/L, which was statistically higher than that of females (46.7 (34.4–60.9) nmol/L) (*P* < 0.001). The general prevalence of vitamin D deficiency was 53.2%; 50.0% for males and 56.5% for females at the cut-off 50 nmol/L. According to the results of the log-binomial regression analysis, vitamin D deficiency in Chinese children and adolescents was specifically related to female gender (*P* < 0.0001), to ages 12–14 years (*P* < 0.0001) and 15–17 years (*P* < 0.0001), living in large cities (*P* < 0.0001) or ordinary rural areas (*P* < 0.0001), low ambient UVB levels (*P* < 0.0001) and medium ambient UVB levels (*P* < 0.0001), spring (*P* < 0.0001), autumn (*P* < 0.0001) and winter seasons (*P* < 0.0001). The data showed that vitamin D deficiency was very common among children and adolescents aged 6–17 years in China. Effective sun exposure should be encouraged in both genders aged 6–17 years, dietary vitamin D and vitamin D supplements are also recommended, especially in the seasons of spring and winter.

## 1. Introduction

Vitamin D (calciferol) is a fat-soluble hormone and helps ensure the body absorbs and retains calcium and phosphorus. Both elements are the cornerstones of a healthy body, and they are essential for bone mineralization. Without vitamin D, only 10–15% of dietary calcium can be absorbed [1]. There is increasing evidence showing that vitamin D may also have potential benefits for cardiometabolic risk factors, immunity, and cancer prevention [2]. Vitamin D deficiency can lead to rickets, a bone-softening disease which also increases the risk of bone fractures in children, teenagers, and adults. 25-hydroxyvitamin-D (25(OH)D) is the major circulating form of vitamin D in the blood with relatively higher concentration in blood, and it was considered as the most reliable biological indicator for vitamin D status assessment [3]. Therefore, screening for vitamin D deficiency by obtaining a serum 25(OH)D concentration is recommended.

As a result of its potential relationship with many diseases and physical conditions [1], there has been an increasing interest in knowledge of the status of vitamin D, not only from scientists, but also the public. Childhood and adolescence are two rapid growth periods of life, and they need a variety of nutrients, among which vitamin D is beneficial for the normal development of bones. However, the vitamin D status of Chinese children and adolescents has not been assessed so far. In the year of 2010–2012, the serum 25(OH)D concentrations were firstly measured in our laboratory and became a continuous monitoring indicator in the Chinese national nutrition and health survey(CNNHS). 

Serum 25(OH)D is a good indicator of vitamin D stores and is the optimal method of assessing vitamin D status [2]. Therefore, the nutritional status of vitamin D was evaluated by serum 25(OH)D, but the cut-off levels for vitamin D deficiency is still an area of controversy [4,5,6]. We adopted the cut-off levels for sufficient vitamin D recommended by the American Institute of Medicine (IOM) [7] and the European Society for Paediatric Gastroenterology, Hepatology and Nutrition [8]. 

The objective of this study is to understand the vitamin D status of Chinese children and adolescents aged 6–17 years by detecting serum 25(OH)D concentration, and to evaluate the vitamin D status of them. This is critical for formulating corresponding intervention policy advice for the population groups.

## 2. Materials and Methods 

### 2.1. Subjects and Ethics

Survey participants aged 6–17 years old came from CNNHS (2010–2012). CNNHS is a cross-sectional survey of the civilian non-institutionalized population of China, conducted by the National Institute of Nutrition and Health, Chinese Center for Disease Control and Prevention (NINH, Chinese Center for Disease Control and Prevention). All the participants of this survey were selected by using a stratified proportional random cluster sampling. All county (district) level administrative units (including counties, county-level cities and districts) from 31 provinces of the Chinese mainland were divided into four categories, namely large cities, small and medium-sized cities, rural areas and poor rural areas. During the first stage, 34 large cities, 41 small and medium-sized cities, 45 rural areas and 30 poor rural areas were selected as investigation points by a simple random sampling method. Then, during the second stage, six village committees or neighborhood committees from each point were selected by the population proportional sampling method. In the last stage, 75 households from each selected village or neighborhood committee were selected by a simple random sampling method. The number of children and adolescent participants for serum 25(OH)D detection from each monitoring point should be up to 100; if not, we recruited participants from nearby schools to supplement the sample size. A total of 15,000 children and adolescents were investigated and had blood collected for serum 25(OH)D detection (See Figure S1). All participants and their guardians gave their informed consent in writing to participate in the study. The Ethics Review Board of the Institute for Nutrition and Health, China CDC approved the protocol (2013-018). 

### 2.2. Blood Sample and 25-Hydroxyvitamin D Measurement

A national project workgroup was established in NINH, China CDC to develop a unified survey and questionnaire to carry out the investigation survey by using unified equipment and methods. The basic information of the subjects (including age, gender, city type, outdoor activities, etc.) was collected by questionnaire. 

2 mL of fasting venous blood was collected and centrifuged at 1500× *g* for 15 min within 30 min after the blood was taken. The upper serum was aliquot and stored in a brown vessel at −20 °C in the laboratory where the investigation point was located. All the serum specimens from all the 150 investigation points were transported to our laboratory by cold chain. All the samples were preserved at −70 °C in a refrigerator before determination. Serum 25(OH)D concentration were measured in our laboratory by 25(OH)D radioimmunoassay kits (DiaSorinRIA; DiaSorin Inc., Stillwater, MN, Canada). The interassay coefficients of variation (CVs) were 3.8% and 3.5% at 24.8 and 57.5 nmol/L, respectively. 

### 2.3. Criteria of Vitamin D Deficiency 

A serum 25(OH)D concentration of 50 nmol/L(20 ng/mL) is considered sufficient [5,7,8]. This value was based on the assumption of minimal exposure to sunlight and minimal solar vitamin D conversion. The serum 25(OH)D concentration which was lower than 50 nmol/L was considered deficient, and lower than 25 nmol/L was considered to be severely deficient [8].

### 2.4. Variables

All the information regarding participants in this study was collected from every investigation point and logged into the systematic platform of the national survey of nutrition and health status for Chinese residents. Region type was recorded and classified [9]. Based on a self-report, demographic data (including age, gender, ethnicity) and the types and frequency use of vitamin D supplementation were recorded. Outdoor time was also recorded, because sufficient sun exposure could effectively promote the transformation of vitamin D [3]; therefore, enough outdoor activity time with direct exposure to the sun was another important factor. The season was recorded according to the month of blood collection. China’s Qinling Mountains and Huaihe River are recognized as the boundary to divide the north and the south. According to the latitude and longitude information of the monitoring area, the corresponding dose of erythematic-weighted ultraviolet radiation B (UVB) level was obtained by searching the National Aeronautics and Space Administration of America (NASA) database. The ambient UVB radiation exposure level for each participant was then recorded. The doses were classed into three grades, the annual mean value of UVB, where a value lower than 2900 J/m^2^ was defined as low, 2900–3750 J/m^2^ was defined as medium, and higher than 3750 J/m^2^ was defined as high [10,11]. Body Mass Index (BMI) was calculated according to bodyweight and height. Body weight was measured by using a double lever weight scale, and standing height was measured by a metal column type height meter. Overweight and obesity were defined by using cut points for different age and gender recommended by WHO [12] and Group of China Obesity Task Force [13].

### 2.5. Data Analyses

All the data was analyzed by SAS 9.4 software (SAS Institute Inc., Cary, NC, USA). All the participants in this study were divided into different sub-groups according to the different hypothesized predictors for vitamin D status. Serum 25(OH)D concentrations were recorded by using P50 (P25–P75) because they were not consistent with the normal distribution according to the normality test, and then they were compared by the Kruskal–Wallis test. Frequencies were presented as percentages (%) and the rates of subgroups were compared by the chi square test. If the frequency was less than five or the cases were less than 40, Fisher’s exact test was used.

The odds ratio by logistical regression can overestimate the prevalence ratio in cross-sectional studies, especially when working with frequent outcomes (above 10%) [14,15]. Therefore, we adopted a prevalence ratio (PR) calculated by the log-binomial regression method to estimate the risk of potential related factors for vitamin D deficiency instead of logistical regression. The log-binomial regression analysis was utilized to analyze the relationship between vitamin D deficiency and possible predictors (e.g., age, gender, region type, latitude, ambient UVB level at area of residence, season, BMI, and outdoor time). The PR and 95% confidence intervals (CIs) were determined by the log-binomial regression model [14]. The difference was statistically significant with *P* < 0.05.

## 3. Results

Excluding those with severe hemolytic serum or unqualified data, the serum 25(OH)D level of 14,473 healthy individuals (7288 males and 7185 females) aged 6–17 were analyzed in this study (Table 1). The median age of children and adolescents was 12.1 years old (interquartile range (IQR) 9.1–14.9, range 6.0–17.9). Age groups were classified according to different learning stages; there were 7037 children aged 6–11 (primary school), 3928 adolescents aged 12–14 (junior high school) and 3508 adolescents aged 15–17 (high school). Sufficient sun exposure could effectively promote the transformation of vitamin D [3]; therefore, enough outdoor activity time with direct exposure to the sun was another important factor. In this study, we classified outdoor activity time into three grades: less than 30 min (68.2%), 30–60 min (20.1%) and more than 60 min (11.9%). Season was also related to violate radiation; this is another factor for vitamin D in vivo synthesis, and our blood was taken over four seasons: spring (1003, March to May), summer (847, June to August), autumn (7948, September to November) and winter (4675, December to February). 

The concentration of 25(OH)D for all participants of different sub-groups is presented in Table 1. The median serum 25(OH)D is 48.2 nmol/L (IQR 35.4–63.4). Median serum 25(OH)D concentrations were significant higher in males than in females (*P* < 0.001). Median serum 25(OH)D concentrations differed by age; they were highest in children aged 6–11 and significantly lower in each succeeding age group. Median serum 25(OH)D concentrations of participants from large cities were significantly lower than from other regions (*P* < 0.001). The 25(OH)D concentration of the participants from the south was significantly higher than those from the north (*P* = 0.0015). Those who had more than 60 min of outdoor activities had higher 25(OH)D concentration. Participants from a high-level UVB radiation area also had higher 25(OH)D levels. Median serum 25(OH)D concentration were highest in participants whose blood was taken in summer and lowest in those whose blood was taken in spring. No significant difference was found between the Han ethnic group and other ethnic groups (*P* = 0.5870), and no significant difference was found between different BMI levels (*P* = 0.0654).

The prevalence of vitamin D deficiency was 53.2% (7.2% for severely deficient) in Chinese children and adolescents in 2010–2012 (Table 2). A higher prevalence of deficiency was significant in females (56.5%) rather than in males (50.0%, *P* < 0.001). The prevalence of vitamin D deficiency was gradually increased by age both in males and females. It was significantly lower in succeeding age groups in males, and it was significantly lower at ages 6–11 than 12–17. Small and middle-sized cities had the lowest prevalence of vitamin D deficiency in females (53.8%), and poor rural areas had the lowest prevalence in males (46.5%). The male participants from the north of China have a significantly higher prevalence of vitamin D deficiency than those from the south. The prevalence of vitamin D deficiency for those whose blood was taken in summer was significantly lower than those whose blood was taken in any other season. The vitamin D deficiency prevalence decreased by the increase of UVB level and outdoor time. As for the ambient UVB level, a high level of UVB radiation significantly decreased the prevalence of vitamin D deficiency in both males and females. Those whose daily outdoor activity was less than 30 min had a significant higher prevalence of vitamin D deficiency than those who had more than 30 min in males, and lowest prevalence of vitamin D deficiency was showed in those who had more than 60 min outdoor activity in females. The prevalence of vitamin D deficiency in the Han ethnic group was higher than that of other ethnic group (*P* = 0.0123).

In the log-binomial regression analysis for all the participants, we analyzed factors including gender, age group, region type, ethnic group, latitude, ambient UVB level, season, outdoor activity time and BMI (Table 3). The results of the log-binomial regression analysis showed that the vitamin D deficiency was most strongly associated with females (PR, 1.31, *P* < 0.0001, relative to males) and ages 12–17 (ages 12–14, PR 1.61, *P* < 0.0001; ages 15–17, PR 1.72, *P* < 0.0001; relative to those aged 6–11). Besides this, participants from large cities or ordinary rural areas had an increased risk of vitamin D deficiency; the PR were 1.37 (*P* < 0.0001) and 1.34 (*P* < 0.0001), respectively. Ambient UVB values of lower than 3750 J/m^2^ increased the risk of vitamin D deficiency (low, PR 1.59, *P* < 0.0001; medium, PR 1.39, *P* < 0.0001; relative to high ambient UVB level). Taking summer as the reference standard, the other seasons all increased the risk of vitamin D deficiency, and the PR were 2.59(*P* < 0.0001) for spring, 1.94 (*P* < 0.0001) for autumn and 2.82 (*P* < 0.0001) for winter. A similar tendency was found in both genders in terms of age groups, region types, ambient UVB levels and season according to the log-binomial analysis of males and females, respectively. Though significant differences were found in different ethnic groups, latitude, and outdoor activity time with regard to the prevalence of vitamin D deficiency, no significant difference was found in the risk of vitamin D deficiency in different sub-groups.

## 4. Discussion and Conclusions

In the CNHHS 2010–2012 survey, we determined serum 25(OH)D concentration for the first time by utilizing the diasorin-Radioimmunoassay (RIA) method. The diasorin-RIA method does not need complicated sample pretreatment and purification processes, which had advantages for large sample size detection. Some studies reported that the RIA method had a good correlation with the combination of liquid chromatography with tandem mass spectrometers LC-MS/MS method (gold standard) in 25(OH)D determination. Ouweland et al. [16] found that the diasorin-RIA agreed well with the LC-MS/MS method (*r^2^* = 0.90; average bias 1.61 nmol/L) by detecting room quality control specimens from the international vitamin D room quality assessment program (DEQAS). We also made a comparison between the diasorin-RIA method and the LC-MS/MS method. The results showed that the correlation coefficient between diasorin-RIA and LC-MS/MS was 0.82, indicating that diasorin-RIA was in acceptable agreement with LC-MS/MS in the assessment of vitamin D deficiency [10].

In 2010–2012, the median concentration of Chinese children and adolescents aged 6–17 was 48.2 nmol/L, which was considerably lower than that of the elderly Chinese population (61 nmol/L) [17]. The concentration of serum 25(OH)D decreased by latitude and age, and a similar trend in age was also found in the National Health and Nutrition Examination Survey of America (NHANES) 2001–2006 [18]. The concentration of 25(OH)D increased by outdoor time and ambient UVB level. Children who had the longer outdoor activities time in our study had higher 25(OH)D levels. 

The prevalence of vitamin D deficiency was up to 53.2% by the cut-off 50 nmol/L serum 25(OH)D, 50.0% in males and 56.5% in females in Chinese children and adolescents in 2010–2012. The prevalence was higher than children aged 1–11 (9.7%) and aged 12–19 (25.0%) in America during 2007–2010 from NHANES [19], children aged below 19 in Canada (29.4% for males aged 9–13; 35.7% for males aged 14–18; 33.0% for females aged 9–13 and 33.7% for females aged 14–18) from the Canadian Health Measures Survey during 2007–2011 under the same criteria [20]. The deficiency of children and adolescents was higher than that of elderly men (34.1%) and women (44.0%) in China, respectively [17]. 

Children aged 12–14 and 15–17 had an increased risk of vitamin D deficiency, possibly due to the gradually increased schoolwork which occupied the time for outdoor activities. The highest prevalence was found in spring, and the lowest prevalence was found in summer. Female participants had the highest rate (65.0%) of vitamin D deficiency prevalence in spring, and male participants had the lowest rate (34.6%) of vitamin D deficiency prevalence in summer. Similar seasonal differences were observed in children and adolescents from southeastern China in all age groups [21]. The seasonal factors and gender were also found in the US children from NHANES during 2007–2010. Less sun exposure for females might be one of the reasons why female children had a 1.31-fold increased risk of vitamin D deficiency compared to males. Children living in large cities and ordinary rural areas are more at risk for vitamin D deficiency than those live in small and medium-sized cities and poor rural areas. Air pollution in the cities can decrease the ambient UVB level and affect the vitamin D health of the urban population [22]. Sunscreen use in children and adolescents, which protects against future melanoma, might be another reason for decreased vitamin D production in the skin [23]. This might explain why children from large cities had an increased risk. Another reason why children in large cities had the highest deficiency is probably due to a lack of outdoor activities in both genders. Sunlight exposure is the primary determinant of serum 25(OH)D levels in humans [24], which was also proved in our study. The result of this study showed that season and UVB level were strongly associated with vitamin D status according to the result of the multivariate logistic regression analysis. Turer et al. [5] reported that overweight and obese children had significantly greater odds of vitamin D deficiency compared with healthy weight children aged 6–18 from NHANES 2003–2006. However, compared to children with normal BMI, no significant difference was showed in overweight and obese children in terms of 25(OH)D concentration and vitamin D deficiency.

The authors acknowledge several limitations. We were unable to determine the duration of sun exposure for each participant. We adopted the ambient UVB levels to estimate the sun exposure levels for all the participants, and the results showed that these ambient UVB levels affect the serum 25(OH)D levels among Chinese children and adolescents. Unfortunately, dietary sources of vitamin D and calcium intake levels, which were considered as important factors to the serum 25(OH)D levels [25], were not estimated for each participant. Vitamin D supplements were investigated in our questionnaires, but there are very few people taking supplements (203/14,473, 1.40%). The results showed no statistical difference observed in terms of 25(OH)D concentration. However, the disparity in data may lead to bias. 

In summary, the problem of vitamin D deficiency in Chinese children and adolescents is very serious; more than 50% children and adolescents in China in our study were vitamin D deficient. The public should pay more attention to children and adolescents' nutrition status. Most children and adolescents did not get enough sunlight, particularly during spring and winter; with the increase of UVB radiation, the risk of vitamin D deficiency was decreased. Thus, for children and adolescents with vitamin D deficiency, sunscreen use should be reduced, and they should be encouraged to partake in more outdoor activities. Dietary vitamin D or vitamin D supplements are also recommended under conditions of insufficient sun exposure caused by seasonal and clothing issues, especially in spring and winter.

## Figures and Tables

**Table 1:** 25(OH)D concentration for children and adolescents from the China Nutrition and Health Survey 2010–2012.

Variables	*N* (%)	25(OH)D ^1^ (nmol/L)	*P* Value ^2^
Total	14,473	48.2 (35.4–63.4)	
Gender			<0.0001
Male	7288 (50.36)	50.0 (36.5–65.7)	
Female	7185 (49.64)	46.7 (34.4–60.9)	
Age group			<0.0001
6–11	7037 (48.62)	51.6 (38.1–67.3)	
12–14	3928 (27.14)	45.5 (33.3–60.4)	
15–17	3508 (24.24)	44.3 (33.3–585)	
Region type			<0.0001
Large cities	3188 (22.03)	46.1 (34.4–61.0)	
Small and medium-sized cities	4514 (31.19)	49.7 (36.8–65.0)	
Ordinary rural area	4015 (27.74)	47.7 (33.6–63.7)	
Poor rural areas	2756 (19.04)	49.6 (36.1–63.3)	
Ethnicity			0.5870
Han	12,867 (88.90)	48.1 (35.6–63.6)	
Other	1606(11.10)	49.3 (36.0–62.6)	
Latitude			0.0015
North	6778 (46.83)	47.2 (34.8–62.4)	
South	7695 (53.17)	49.1 (35.9–64.2)	
Season			<0.0001
Spring	1003 (6.93)	44.1 (35.4–59.7)	
Summer	847 (5.85)	57.1 (43.8–73.1)	
Autumn	7948 (54.92)	49.6 (35.8–61.2)	
Winter	4675 (32.30)	45.2 (26.5–59.1)	
Ambient UVB ^3^ level (J/m^2^)			<0.0001
Low	4742 (32.77)	47.0 (34.3–60.1)	
Medium	5018 (34.67)	46.8 (34.7–62.1)	
High	4713 (32.56)	50.8 (37.4–65.9)	
Outdoor time			<0.0001
Low than 30 min	9865 (68.16)	47.6 (34.9–62.9)	
30~60 min	2897 (20.02)	48.9 (36.1–64.4)	
More than 60 min	1711 (11.82)	49.9 (37.3–64.9)	
BMI ^4^			0.0654
Normal	12,022 (83.07)	48.0 (35.2–63.5)	
Overweight	1476 (10.20)	48.3 (35.4–61.9)	
Obesity	975 (6.73)	49.6 (37.0–64.5)	

^1^ P50 (P25–P75), all such values; ^2^ *P* value for the Kruskal–Wallis test. ^3^ UVB– ultraviolet radiation B; ^4^ BMI-Body mass index.

**Table 2:** Prevalence of vitamin D deficiency oinChinese children and adolescents in the Chinese nutrition and health survey 2010–2012 (%).

Variables	Male				Female				Total			
	Sufficient	Deficient		*P* Value	Sufficient	Deficient		*P* Value	Sufficient	Deficient		*P* Value
	≥50	25–50	<25		≥50	25–50	<25		≥50	25–50	<25	
Total	50.0	43.2	6.8		43.5	48.9	7.6		46.8	46.00	7.2	
Age group				<0.0001				<0.0001				<0.0001
6–11	55.7	39.4	4.9		50.9	42.9	6.2		53.3	41.1	5.6	
12–14	46.1	44.9	9.0		37.0	54.3	8.7		41.6	49.6	8.8	
15–17	42.7	48.9	8.4		36.2	54.8	9.0		39.5	51.8	8.7	
Region type				<0.0001				<0.0001				<0.0001
Large cities	44.9	46.4	8.7		38.9	50.9	10.2		41.9	48.7	9.4	
Small and medium-sized cities	52.1	43.5	4.4		46.2	48.8	5.0		49.2	46.1	4.7	
Ordinary rural area	49.1	41.1	9.8		43.1	46.7	10.2		46.1	43.9	10.0	
Poor rural areas	53.5	41.9	4.6		45.1	49.8	5.1		49.3	45.9	4.8	
Ethnic group				0.0187				0.1022				0.0123
Han	49.47	43.46	7.07		43.6	48.6	7.8		46.5	46.00	7.5	
Other	54.01	40.89	5.10		43.2	50.9	5.9		48.5	46.0	5.5	
Latitude				<0.0001				0.0028				<0.0001
North	48.3	45.9	5.8		42.1	50.9	7.0		45.2	48.4	6.4	
South	51.5	40.7	7.8		44.8	47.1	8.1		48.1	43.9	8.0	
Season				<0.0001				<0.0001				<0.0001
Spring	45.6	49.3	5.1		35.0	59.1	5.9		40.4	54.1	5.5	
Summer	65.4	31.1	3.5		59.7	36.5	3.8		62.6	33.8	3.6	
Autumn	52.3	40.4	7.3		46.0	46.1	7.9		49.2	43.2	7.6	
Winter	44.1	48.9	7.0		38.2	53.6	8.2		41.2	51.2	7.6	
Outdoor time				0.0409				0.0398				0.0395
Low than 30 min	49.0	43.8	7.2		42.9	49.5	7.6		45.9	46.7	7.4	
30~60 min	52.3	41.4	6.3		43.3	48.9	7.8		47.9	45.1	7.0	
More than 60 min	51.7	42.4	5.9		47.5	45.1	7.4		49.6	43.8	6.6	
Ambient UVB level (J/m^2^)				<0.0001				<0.0001				<0.0001
Low	47.6	46.3	6.1		41.7	50.4	7.9		44.7	48.3	7.0	
Medium	47.8	44.9	7.3		40.6	52.2	7.2		44.2	48.6	7.2	
High	54.6	38.1	7.3		48.4	44.0	7.6		51.5	41.0	7.5	
BMI				0.0126				0.8588				0.0892
Normal	50.0	43.0	7.0		43.5	48.8	7.7		46.7	45.9	7.4	
Overweight	47.9	44.2	7.9		44.4	49.0	6.6		46.3	46.4	7.3	
Obesity	52.3	44.0	3.7		42.8	49.7	7.5		48.9	46.0	5.1	

**Table 3:** Determinants of vitamin D deficiency among Chinese children and adolescents from the Chinese nutrition and health survey 2010–2012 ^1^ (log-binomial regression).

	Total (*n* = 14,473)		Male (*n* = 7288)		Female (*n* = 7185)	
	PR	*P* Value	PR	*P* Value	PR	*P* Value
Gender						
Male	ref					
Female	1.31 (1.22–1.40)	<0.0001				
Age group						
6–11	ref		ref		ref	
12–14	1.61 (1.48–1.74)	<0.0001	1.47 (1.31–1.64)	<0.0001	1.77 (1.58–1.99)	<0.0001
15–17	1.72 (1.59–1.88)	<0.0001	1.65 (1.46–1.85)	<0.0001	1.83 (1.62–2.06)	<0.0001
Region type						
Small and medium-sized cities	ref		ref		ref	
Large cities	1.37 (1.24–1.50)	<0.0001	1.35 (1.18–1.54)	<0.0001	1.38 (1.21–1.58)	<0.0001
Ordinary rural area	1.34 (1.22–1.46)	<0.0001	1.33 (1.17–1.50)	<0.0001	1.35 (1.19–1.54)	<0.0001
Poor rural areas	1.04 (0.94–1.16)	0.4181	1.01 (0.88–1.17)	0.8521	1.08 (0.93–1.24)	0.3318
Ethnic group						
Han	ref		ref		ref	
Other	1.00 (0.89–1.12)	0.9609	0.92 (0.78–1.09)	0.3312	1.08 (0.92–1.27)	0.3484
Latitude						
South	ref		ref		ref	
North	1.07 (0.95–1.20)	0.2958	1.05 (0.89–1.24)	0.5799	1.09 (0.92–1.29)	0.3396
Season						
Summer	ref		ref		ref	
Spring	2.59 (2.13–3.15)	<0.0001	2.40 (1.82–3.16)	<0.0001	2.87 (2.17–3.80)	<0.0001
Autumn	1.94 (1.66–2.25)	<0.0001	1.91 (1.54–2.37)	<0.0001	1.99 (1.610–2.47)	<0.0001
Winter	2.82 (2.40–3.31)	<0.0001	2.75 (2.20–3.46)	<0.0001	2.94 (2.34–3.68)	<0.0001
Outdoor activity time						
>60 min	ref		ref		ref	
<30 min	1.05 (0.94–1.16)	0.4193	0.99 (0.86–1.15)	0.9205	1.10 (0.95–1.28)	0.2229
30–60 min	1.06 (0.93–1.19)	0.3856	0.96 (0.81–1.14)	0.6065	1.17 (0.98–1.39)	0.0788
Ambient UVB Level						
High	ref		ref		ref	
Low	1.59 (1.38–1.84)	<0.0001	1.55 (1.27–1.90)	<0.0001	1.64 (1.33–2.02)	<0.0001
Medium	1.39 (1.27–1.53)	<0.0001	1.33 (1.17–1.52)	<0.0001	1.46 (1.28–1.67)	<0.0001
BMI						
Normal	ref					
Overweight	1.01 (0.90–1.13)	0.8970	1.11 (0.96–1.29)	0.1621	0.87 (0.73–1.03)	0.0980
Obesity	0.98 (0.86–1.12)	0.4787	0.90 (0.76–1.07)	0.2425	1.13 (0.91–1.41)	0.2635

^1^ Univariable logistic regression.

## Supplementary Materials

The following are available online at www.mdpi.com/2072-6643/9/9/1024/s1, Figure S1: Participant flow chart.
