# Supplementary material for: Vitamin D Nutritional Status and Its Related Factors for Chinese Children and Adolescents in 2010–2012
Source: Nutrients. 2017 Sep 15;9(9):1024. doi: 10.3390/nu9091024 (PMC5622784; doi:10.3390/nu9091024)
Supplement: Supplementary file 1 [file nutrients-09-01024-s001.zip › nutrients-216295-supplementary.pdf]

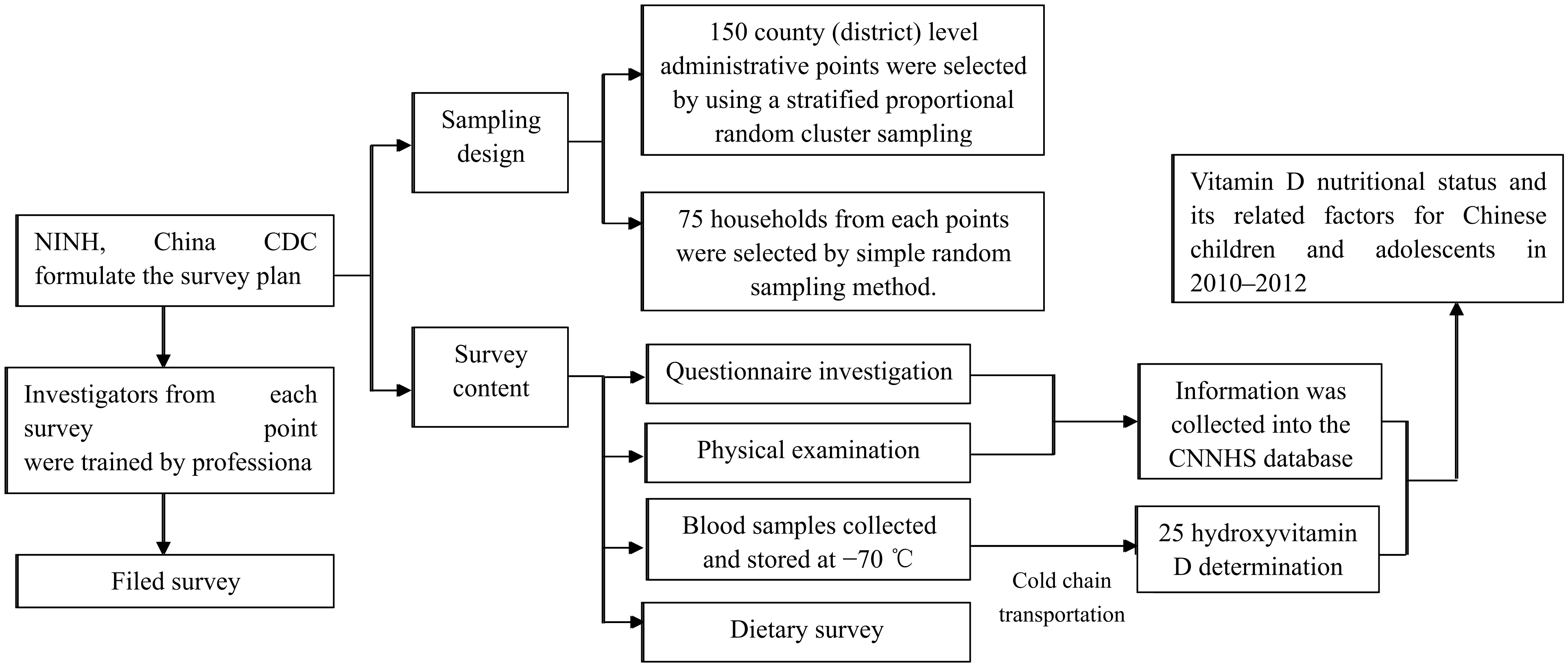

Figure S1. Flow chart for the study of vitamin D nutritional status and its related factors for Chinese children and adolescents in 2010–2012**.**
